# Mr. Leeson, on Croup

**Published:** 1800-07

**Authors:** B. Leeson

**Affiliations:** Grantham


### *To the Editors of the Medical and Phyfical Journal.*

GENTLEMEN,

SHOULD the following obfervations appear worthy your attention, you will much oblige me by inferting them in your ufeful Journal. I am,

Your's,

*Grantham, May* 10, 1800. B. LEESON, jun.

---

It is much to be lamented, that, in the narration of medical facts, an unjuft preference is given to fuch as have had a favourable iffue; while unfuccefsful cafes, however interefting in their progrefs, or important in their event, feldom are brought before the public eye. The felf-gratification which occafions this partiality to fuccefsful cafes, leads alfo to another error, ftill more injurious to found practice and medical improvement,—to the publication of fortunate cafes, the very exiftence of which is doubtful. Not that I can fuppofe any medical practitioner fo void of principle, as wilfully to impofe upon the public, fictitious cafes; but, I doubt not, it will be granted, an attachment to a preconceived opinion will frequently prevent a proper attention to the difcrimination of difeafe. Hence, complaints of the moft ferious import are frequently reported to have yielded to remedies of trifling efficacy; which, if again employed, on the authority of fuch reports, are only productive of difappointment. I have been led to thefe reflections by fome accounts I have lately feen, of the fuccefsful treatment of the croup. Judging from thefe defcriptions, a perfon would naturally conclude the croup to be a difeafe of long duration and eafy management. As by one author we are informed, that mercury employed, fo as to produce falivation, effectually cures: another is confident of the fuccefs of a lotion made with the fpiritus ætheris vitriolici compofitus; while a third relies upon a decoction of feneka.

No

No doubt, all thefe remedies might be productive of good effect, if the rapid progrefs of the complaint allowed them to be fairly tried. But I am afraid, fuch is the celerity of the dangerous fymptoms, that few practitioners have had the pleafure to experience a recovery from the true croup. It may be right, I fhould define what I underftand by the *true croup*: By this term, then, I would exprefs a difeafe, arifing from an extravafation of coagulable lymph within the trachea and bronchial tubes, which occafions that peculiar found in infpiration we fhould expect, was the breath drawn through a narrow pipe. This is preceded by a flight inflammatory ftage, of which the fymptoms are fo little troublefome, as feldom to be obferved.

In confidering the cynanche trachealis, it is neceffary to premife, that two diftinct difeafes appear to have been claffed by writers under the fame name. The one arifing from a fpafmodic ftricture of the parts furrounding the trachea; the other depending on extravafation, the confequence of inflammation. In the firft, the exhibition of an emetic feldom fails to remove the complaint, while the fecond bids defiance to every effort of art. In the fpafmodic croup, the attack is fudden, generally commencing fome time after the patient has been in bed; it is accompanied by remarkable anxiety, and oppreffion about the breaft; a hoarfe, fhrill voice; great rednefs of the countenance; (which expreffes moft grievous uneafinefs) quick and difficult refpiration, and a foft pulfe. Upon the operation of an emetic, thefe fymptoms gradually fubfide; the patient finks into a flumber, and awakes with little remains of the complaint. I am acquainted with a family, in which this complaint has attacked more than once, each child of a numerous offspring, and has never failed to difappear upon the operation of an emetic.

Very different is the progrefs of the inflammatory croup; in this, the firft appearance of difeafe is of fuch an infidious nature, as feldom to create any alarm, being confidered by the attendants as a flight cough accompanied with hoarfenefs. By degrees the roughnefs of the voice becomes more remarkable, the breath is drawn with difficulty, as if through a narrow pipe, occafioning a peculiar fhrill found;* there is a conftant feverifh heat upon the fkin, together with a profufe perfpiration about the head and face, in fo much that the fweat ftands in drops upon the countenance, which exhibits the greateft anxiety; the pulfe is quick and foft; the lips are pale,

* Febris excitatur ad liberandum corpus a muco, & membrana extra vafa.
*Home de Suffocatione Stridula.*

frequently livid; the changes of countenance are fudden and frequent; at one time it is red, in an inftant it is pale as a corpfe. The progrefs of this difeafe, from its commencement to its termination, as far as you may depend upon the information of nurfes, never exceeds more than four or five days: from the firft appearance of danger, the patient feldom continues more than thirty-fix hours, rarely fo long. I have been obliged to refer to nurfes, as I believe few medical men have witneffed the firft attack of croup, it being too inconfiderable to merit their attention.

Having endeavoured to enumerate the pathognomonic fymptoms of the croup, I fhall farther intrude by detailing two cafes which have lately occurred to me.

G. M. eleven months old, naturally of a full habit, recently weaned, and now about his teeth; as he has generally had a cough and ftuffing while cutting his teeth, the nurfe was not alarmed at this circumftance, which had occurred for a day or two before I faw him. I was firft called about eight o'clock in the evening; the great anxiety, difficulty of breathing, and peculiar found in refpiration, clearly indicated his complaint to be the croup: his gums were lanced, an emetic mixture, compofed of four grains of emetic tartar, one drachm of oxymel of fquills, and an ounce and a half of water, was given in dofes of two tea fpoons full every ten minutes until it operated; a lotion, compofed of fp. ætheris vitriolici compofitus, and the aqua ammoniæ acetata, was applied to the throat. At nine o'clock, the fymptoms continuing equally urgent, I had the affiftance of an eminent phyfician refident in this town; by his advice, leeches were applied to the throat, and the patient put into a warm bath; blifters were likewife laid on each fide the neck; from thefe means fome relief appeared to be gained. At eleven o'clock, the child being more reftlefs, was again immerfed in warm water; an oily mixture was given occafionally. At four o'clock in the morning, the violence of the fymptoms increafing, an ounce of ipecacuanha wine was given, in fmall quantities, before it produced any effect; the warm bath was again ufed; about feven o'clock the child expired.

April 20, J. L. aged twenty-two months, yet ftill at the breaft, has had a flight cough for a few days; it has increafed much during the night; the child has been very reftlefs, and fweats much about the face and head; fwallows with tolerable eafe; breathes with much anxiety, and with a peculiar fhrill found. It was nine o'clock in the morning when I firft faw this child; being aware, from the fatal termination of the former cafe, of the neceffity of powerful means to arreft the progrefs progrefs of the diforder, I immediately opened the jugular veins, and obtained from thence between fix and feven ounces of blood; after which a folution of fix grains of emetic tartar in an ounce and a half of water, with a drachm of oxymel of fquills, was given in dofes of two tea fpoons full every ten minutes; the whole mixture was given before any vomiting was produced; the child was then placed in the warm bath for feven minutes. For a fhort time it appeared to be more compofed, and to breathe with lefs difficulty; but, about twelve o'clock, the former fymptoms returned. A tea fpoon full of the decoction radicis feneka was then given every half hour, which excited great thirft, and additional reftleffnefs; the patient grew worfe, breathed with more and more difficulty, and expired about three o'clock P. M.

It has generally been obferved, that the croup is moft prevalent during a wet feafon, or in damp fituations; thefe two cafes occurred when the weather was more than ufually dry.

It will be obferved in the above cafes, that there was confiderable diminution of the fenfibility of the ftomach; as appears from the quantity of emetic medicine neceffary to produce vomiting. May not this arife from an increafed determination of blood to the trachea, diminifhing the influx in the veffels of the ftomach?

